# Impact of Anti-tumor Necrosis Factor (Anti-TNF) Therapy on Cardiovascular Risk in Patients With Ankylosing Spondylitis

**DOI:** 10.7759/cureus.91185

**Published:** 2025-08-28

**Authors:** Ahsan Amer, Ahmed Amer, Farzana Hakim, Ahsaan Amer

**Affiliations:** 1 Department of Medicine, Fauji Foundation Hospital, Rawalpindi, PAK; 2 Department of Geriatric Medicine, Royal Bournemouth Hospital, University Hospital Dorset, NHS Foundation Trust, Bournemouth, GBR; 3 Department of Biochemistry, Foundation University Islamabad, Islamabad, PAK; 4 Department of Medicine and Surgery, Al-Nafess Medical College, Islamabad, PAK

**Keywords:** ankylosing spondylitis, anti-tnf therapy, cardiovascular risk, carotid intima-media thickness, inflammation, lipid profile

## Abstract

Background: A chronic inflammatory condition, ankylosing spondylitis (AS), raises the risk of cardiovascular (CV) disease because of ongoing systemic inflammation.

Objective: To evaluate the impact of anti-TNF therapy on CV risk markers and surrogate indicators of subclinical atherosclerosis in patients with AS, and to examine the influence of modifiable lifestyle factors such as smoking, physical activity, and body mass index (BMI) on CV outcomes.

Methodology: This descriptive observational study was conducted at the Department of Rheumatology, Fauji Foundation Hospital, Rawalpindi, from April 2022 to March 2024. The study included 246 patients with AS who had been on anti-TNF medication for at least three months. At baseline, six months, and 12 months, clinical and laboratory parameters such as blood pressure, BMI, lipid profile, erythrocyte sedimentation rate (ESR), and C-reactive protein (CRP) were measured. Doppler ultrasonography was used to measure the carotid intima-media thickness (CIMT) in 197 individuals (80.08%). Multivariate regression was used to account for physical activity, smoking, age, and BMI.

Results: Among 246 patients (189 males, 76.8%; mean age, 41.2 ± 9.7 years), significant reductions were observed over 12 months in inflammatory, lipid, and cardiovascular markers (all *P* < 0.001). CRP decreased from 15.2 ± 5.7 to 5.3 ± 3.9 mg/L, with 177 (71.95%) showing >50% reduction. ESR declined from 38.4 ± 10.6 to 17.4 ± 7.8 mm/hour, with 166 (67.48%) showing >50% reduction. Low-density lipoprotein (LDL) decreased from 131.5 ± 18.7 to 113.4 ± 14.9 mg/dL, with a ≥10% reduction in 153 (62.20%), while high-density lipoprotein (HDL) increased from 38.9 ± 6.8 to 47.2 ± 7.3 mg/dL, with a ≥10% increase in 134 (54.47%). Systolic BP dropped from 136.9 ± 12.2 to 126.8 ± 10.9 mmHg, with a ≥10 mmHg reduction in 121 (49.19%). BMI also declined significantly from 27.6 ± 3.8 to 26.3 ± 3.2 kg/m². Among 197 patients with Doppler ultrasound, CIMT regressed in 142 (72.08%), with right CIMT decreasing from 0.84 ± 0.11 to 0.74 ± 0.09 mm and left CIMT from 0.86 ± 0.10 to 0.75 ± 0.08 mm. Multivariate analysis identified smoking and higher BMI as predictors of adverse changes, while physical activity was protective. Despite these improvements, unhealthy dietary habits remained prevalent, underscoring the need for lifestyle interventions alongside therapy.

Conclusions: Anti-TNF therapy significantly improves inflammatory, lipid, and vascular parameters, thereby reducing CV risk in patients with AS.

## Introduction

The axial skeleton is the primary target of ankylosing spondylitis (AS), a chronic inflammatory rheumatic disease that causes increasing spinal stiffness, discomfort, and functional impairment [1,2]. AS is becoming more well acknowledged as a systemic illness with serious extra-articular consequences, such as increased cardiovascular (CV) morbidity and mortality, in addition to its musculoskeletal symptoms [3]. Endothelial dysfunction, accelerated atherosclerosis, and a higher incidence of conventional CV risk factors such as insulin resistance, dyslipidemia, and hypertension are all exacerbated by the chronic inflammatory load linked to AS [4].

A key proinflammatory cytokine in the pathophysiology of AS, tumor necrosis factor-alpha (TNF-α), is essential for the initiation and maintenance of systemic inflammation [5]. Through processes including oxidative stress, disturbance of lipid metabolism, and activation of vascular endothelium, elevated TNF-α levels have been directly linked to the pathophysiology of atherogenesis [6]. As a result, anti-TNF medication has become a key component in managing AS disease activity and has the potential to influence CV risk [7].

Anti-TNF drugs may have CV preventive benefits by lowering systemic inflammation, enhancing endothelial function, and favorably affecting lipid profiles, according to several observational studies and clinical trials [8,9]. The degree and consistency of these effects are still up for discussion, however, especially in light of differences in patient demographics, comorbidities, length of therapy, and choice of anti-TNF medication [10]. Furthermore, it is unclear how well-established decreases in inflammatory markers like erythrocyte sedimentation rate (ESR) and C-reactive protein (CRP) translate into significant decreases in CV events or surrogate indicators of atherosclerosis [11].

In addition, systemic inflammation plays a critical role by driving endothelial dysfunction, altering cytokine expression, and promoting plaque instability, thereby precipitating acute coronary events. This mechanism is not unique to AS but represents a unifying theme across chronic inflammatory conditions such as inflammatory bowel disease, psoriasis, and chronic kidney disease [12-14].

A better knowledge of the CV ramifications of anti-TNF medication is crucial, especially in light of the rising prevalence of CV illness among AS patients and the developing significance of biologic medicines in modifying systemic inflammatory pathways. This is especially important for people with high baseline CV risk and perhaps restricted access to long-term biologic treatment.

Surrogate indicators of atherosclerosis, such as carotid intima-media thickness (CIMT) and vascular ultrasonography, offer noninvasive ways to detect early vascular remodeling and subclinical disease progression. By integrating these markers with inflammatory and metabolic profiles, the present study provides a comprehensive assessment of CV risk modification.

Research objective

To evaluate the impact of anti-TNF therapy on CV risk markers and surrogate indicators of atherosclerosis in patients with AS. To the best of our knowledge, this represents the first longitudinal evaluation of a Pakistani AS cohort assessing CIMT endpoints in parallel with conventional CV risk factors.

## Materials and methods

Study design and setting

This descriptive observational study was conducted at the Department of Rheumatology, Fauji Foundation Hospital, Rawalpindi, from April 2022 to March 2024, including all eligible patients attending outpatient clinics during this period. The study protocol was approved by the institutional Ethical Committee before initiation, and written informed consent was obtained from all participants before enrollment. Confidentiality and data privacy were strictly maintained throughout the study in accordance with institutional and ethical guidelines.

Inclusion and exclusion criteria

The research study was open to patients who were 18 years of age or older, had been on anti-TNF medication for at least three months before participation, and had a verified diagnosis of AS according to the Modified New York Criteria. A history of CV disease (myocardial infarction, stroke, or heart failure) with a clinical diagnosis, the use of other biologic agents or corticosteroids in excess of maintenance dosages, stage 3 or higher chronic kidney disease, uncontrolled diabetes mellitus, pregnancy or lactation, irregular follow-up, or poor medication adherence were among the exclusion criteria.

Sample size

Convenience sampling was used to recruit 246 patients from the outpatient AS population. The figure of 246 represents the total number of consecutive, eligible patients enrolled over the 24-month recruitment period. This pragmatic approach ensured inclusion of all patients meeting criteria within the defined timeframe, thereby maximizing sample size for greater precision of estimates. The single-center design and the goal of enrolling all consecutive, eligible patients over the research period required the use of convenience sampling. Given that the research was exploratory in nature and sought to replicate actual clinical practice, no formal a priori sample size or power calculation was carried out. However, the final sample size is comparable to that of previous observational studies, such as those by Yönak et al. [15] and Maia et al. [16], which assessed the CV effects of anti-TNF medication in rheumatologic populations. The discussion acknowledges this restriction.

Data collection

Medical record reviews and structured interviews were used to gather clinical and demographic information about the patients. Blood pressure, body mass index (BMI), lipid profile (total cholesterol, low-density lipoprotein (LDL), high-density lipoprotein (HDL), and triglycerides), inflammatory markers CRP and ESR, and biochemical and clinical characteristics were used to assess CV risk. High-resolution Doppler ultrasonography was also used to measure CIMT when it was accessible. All CIMT assessments were performed by the same experienced sonographer, blinded to patients’ clinical and laboratory results, to ensure consistency and minimize measurement bias. Measurements were taken at the distal 1 cm of the common carotid artery, bilaterally, using a standardized protocol. During the course of the research study, baseline measures were obtained upon enrollment, and follow-up evaluations were carried out every six and twelve months. In order to control for possible confounding variables, lifestyle parameters including physical activity, eating habits, and smoking status were also recorded.

Statistical analysis

Data were analyzed using SPSS version 26.0 (IBM Corp., Armonk, NY). Baseline patient characteristics were summarized descriptively: categorical variables were reported as frequencies and percentages (n, %), while continuous variables were presented as means ± standard deviations. Changes in CV risk markers over time (baseline, six months, and 12 months) were assessed using paired-samples t-tests, and the corresponding t-statistics and *P*-values were reported. The assumptions of normality and homoscedasticity were checked using Shapiro-Wilk and Levene’s tests, respectively. Where assumptions were met, parametric tests were applied; otherwise, nonparametric equivalents were considered. Although multiple endpoints were examined, no formal correction for multiple comparisons was performed, given the exploratory nature of the study; this is acknowledged as a limitation. For CIMT, only patients with complete Doppler ultrasound data were included in the analysis. To identify independent predictors of change in CV risk markers at 12 months, multivariate linear regression models were constructed, adjusting for potential confounders, including age, BMI, smoking status, and physical activity. All covariates were entered simultaneously (forced-entry method) rather than stepwise selection, and variables were mean-centered before entry to reduce multicollinearity. Regression coefficients (β), 95% confidence intervals, t-statistics, and *P*-values were reported for each predictor. All statistical tests were two-tailed, and a *P*-value < 0.05 was considered statistically significant.

## Results

Table 1 presents the baseline profile of the study participants. At baseline, the study population of 246 patients was predominantly male, with 189 (76.83%) men and only 57 (23.17%) women. The mean age was 41.2 ± 9.7 years, and the average BMI of 27.6 ± 3.8 kg/m² indicated that most were overweight. Nearly one-third (84, 34.15%) reported smoking, while 162 (65.85%) were non-smokers. Lifestyle assessment showed that physical inactivity was more common, with 135 (54.88%) leading a sedentary lifestyle compared to 111 (45.12%) who were physically active.

**Table 1:** Baseline demographic and clinical characteristics of patients (n = 246).

Category	Variable		Value
Demographics	Age (years)	Mean ± SD	41.2 ± 9.7
	Gender	Male	189 (76.83%)
		Female	57 (23.17%)
Disease characteristics	Duration of AS (years)	Mean ± SD	6.8 ± 3.1
	BMI (kg/m²)	Mean ± SD	27.6 ± 3.8
Lifestyle factors	Smoking status	Smokers	84 (34.15%)
		Non-smokers	162 (65.85%)
	Physical activity	Active	111 (45.12%)
		Sedentary	135 (54.88%)

Table 2 highlights significant reductions in inflammatory and lipid markers over 12 months of anti-TNF therapy. More than two-thirds of patients achieved meaningful decreases in inflammatory markers: 177 (71.95%) showed a >50% reduction in CRP, and 166 (67.48%) achieved a similar reduction in ESR. Lipid changes were also favorable, with 153 (62.20%) experiencing at least a 10% drop in LDL and 134 (54.47%) achieving a comparable rise in HDL, reflecting improved CV risk profiles.

**Table 2:** Changes in cardiovascular risk markers and proportion of patients achieving improvement (n = 246). A paired-samples t-test was used. Proportions of patients achieving meaningful improvements are based on predefined thresholds (e.g., >50% reduction in CRP/ESR, ≥10% change in LDL/HDL, ≥10 mmHg drop in SBP, ≥0.05 mm CIMT reduction). CRP, C-reactive protein; ESR, erythrocyte sedimentation rate; LDL, low-density lipoprotein; HDL, high-density lipoprotein; SBP, systolic blood pressure; CIMT, carotid intima-media thickness

Parameter	Baseline (Mean ± SD)	6 months (Mean ± SD)	12 months (Mean ± SD)	t-value	P-value	Clinically meaningful improvement (% patients)
C-reactive protein (mg/L)	15.2 ± 5.7	8.6 ± 4.2	5.3 ± 3.9	36.2	<0.001	177 (71.95%) >50% reduction
ESR (mm/hour)	38.4 ± 10.6	26.1 ± 9.2	17.4 ± 7.8	31.8	<0.001	166 (67.48%) >50% reduction
Total cholesterol (mg/dL)	204.3 ± 24.5	194.2 ± 21.1	187.9 ± 19.4	18.9	<0.001	-
LDL (mg/dL)	131.5 ± 18.7	120.8 ± 16.2	113.4 ± 14.9	23.4	<0.001	153 (62.20%) ≥10% reduction
HDL (mg/dL)	38.9 ± 6.8	43.5 ± 7.1	47.2 ± 7.3	29.6	<0.001	134 (54.47%) ≥10% increase
Triglycerides (mg/dL)	178.1 ± 33.6	164.9 ± 30.2	154.2 ± 28.8	21.5	<0.001	-
BMI (kg/m²)	27.6 ± 3.8	26.8 ± 3.5	26.3 ± 3.2	12.7	<0.001	-
Systolic BP (mmHg)	136.9 ± 12.2	130.4 ± 11.5	126.8 ± 10.9	21.2	<0.001	121 (49.19%) ≥10 mmHg reduction
Diastolic BP (mmHg)	86.3 ± 8.6	82.7 ± 7.9	79.2 ± 7.4	18.4	<0.001	-
CIMT - Right (mm) (n = 197)	0.84 ± 0.11	0.79 ± 0.10	0.74 ± 0.09	15.3	<0.001	-
CIMT - Left (mm) (n = 197)	0.86 ± 0.10	0.80 ± 0.09	0.75 ± 0.08	16.1	<0.001	142/197 (72.08%) ≥0.05 mm reduction

Over the 12-month period, the mean BMI significantly declined from 27.6 ± 3.8 to 26.3 ± 3.2 kg/m² (*P* < 0.001). Beyond inflammation control, CV risk factors also improved. About half of the cohort (121, 49.19%) experienced a drop of ≥10 mmHg in systolic blood pressure (SBP) over the 12 months of therapy, alongside a significant decline in BMI and diastolic pressure. These findings suggest beneficial effects of anti-TNF therapy on weight and blood pressure regulation in addition to its anti-inflammatory action.

Analysis of the proportion of patients achieving clinically meaningful improvements highlights the broad impact of treatment: 177 participants (71.95%) attained substantial reductions in CRP, and similar trends were noted for ESR, LDL, HDL, and SBP. Importantly, among those evaluated for CIMT, nearly three-quarters demonstrated vascular improvement, underscoring the therapy’s multidimensional benefits.

**Figure 1:**
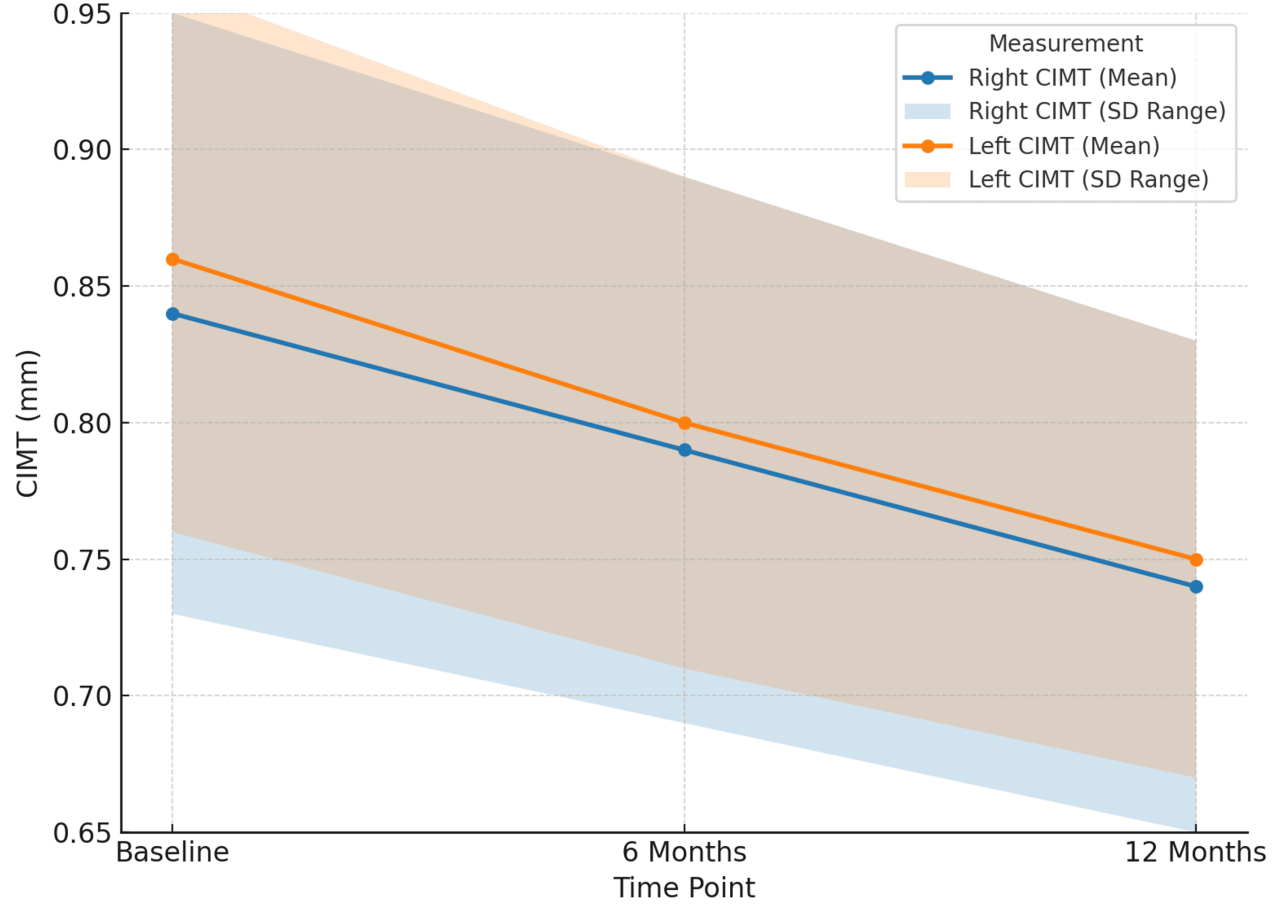
Changes in carotid intima-media thickness (CIMT) over time.

Among the 197 patients with Doppler ultrasound data, CIMT showed meaningful reductions over time (Figure 1). A clear regression of subclinical atherosclerosis was observed: 142 (72.08%) showed measurable CIMT reductions of at least 0.05 mm after 12 months. This supports a potential protective vascular effect of therapy in patients with established inflammatory disease.

Dietary patterns, assessed at baseline only, revealed considerable scope for improvement in CV health behaviors (Table 3). A large proportion of patients (94, 38.21%) reported high intake of saturated fats, and 72 (29.27%) consumed fast food more than three times weekly. In contrast, healthy dietary practices were relatively uncommon, with only 61 (24.80%) reporting high fruit and vegetable intake and just 19 (7.72%) adhering to a Mediterranean-style diet. As these habits were not reassessed during follow-up, their persistence may have limited the full CV benefit of anti-TNF therapy, underscoring the importance of integrating dietary counseling with pharmacologic treatment.

**Table 3:** Distribution of dietary habits among patients at baseline (n = 246).

Dietary pattern	No. of patients (*n*)	Percentage (%)
High saturated fat intake	94	38.21
High fruit and vegetable intake	61	24.8
Frequent fast food consumption (>3/week)	72	29.27
Mediterranean-style diet adherence	19	7.72

Table 4 presents the results of multivariate regression adjusting for confounders. Greater age and BMI were associated with higher CRP and SBP, while physical activity was linked to significant reductions in both. Smoking was a strong predictor of increased CRP (β = 1.67, *P* = 0.001), LDL (β = 2.94, *P* = 0.002), and SBP (β = 4.88, *P* = 0.001). Higher BMI also strongly predicted increases in LDL (β = 0.15, *P* < 0.001) and SBP (β = 0.23, *P* < 0.001), indicating these factors significantly affect CV risk trajectory during anti-TNF therapy.

**Table 4:** Multivariate regression analysis for predictors of change in cardiovascular risk markers (n = 246). β coefficients represent the estimated effect of each independent variable on the dependent variable, adjusted for other variables in the model.

Dependent variable	Independent variable	β (Coefficient)	95% CI (Lower, Upper)	*t*-value	*P*-value
CRP (12-month change)	Age	–0.11	–0.19, –0.04	–3.00	0.003
	BMI	0.09	0.02, 0.16	2.47	0.014
	Smoking	1.67	0.72, 2.63	3.38	0.001
	Physical activity	–1.24	–2.21, –0.27	–2.53	0.012
LDL (12-month change)	Age	0.06	–0.01, 0.13	1.71	0.089
	BMI	0.15	0.09, 0.21	4.91	<0.001
	Smoking	2.94	1.12, 4.77	3.13	0.002
	Physical activity	–2.58	–4.71, –0.45	–2.39	0.018
Systolic BP (12-month change)	Age	0.10	0.01, 0.19	2.21	0.027
	BMI	0.23	0.15, 0.31	5.74	<0.001
	Smoking	4.88	2.11, 7.64	3.43	0.001
	Physical activity	–3.45	–5.92, –0.99	–2.77	0.006

## Discussion

Our study concludes that anti-TNF treatment significantly reduces CV risk factors in patients with AS. Patients had increased inflammatory markers at baseline, with an average ESR of 38.4 ± 10.6 mm/hour and CRP of 15.2 ± 5.7 mg/L. CRP dropped to 5.3 ± 3.9 mg/L and ESR to 17.4 ± 7.8 mm/hour (*P* < 0.001) after a year of anti-TNF treatment, indicating a significant reduction in systemic inflammation. These findings are consistent with prior research, including Saougou et al. [17], who demonstrated similar reductions in CRP with infliximab treatment.

We also observed favorable changes in lipid profiles. LDL declined from 131.5 ± 18.7 to 113.4 ± 14.9 mg/dL, total cholesterol fell from 204.3 ± 24.5 to 187.9 ± 19.4 mg/dL, triglycerides decreased from 178.1 ± 33.6 to 154.2 ± 28.8 mg/dL, and HDL rose from 38.9 ± 6.8 to 47.2 ± 7.3 mg/dL (all *P* < 0.001). Improvements in HDL were particularly notable, given its protective role in atherogenic dyslipidemia associated with chronic inflammation. Compared to other cohorts, our changes in LDL and HDL appear broadly comparable but not greater than those reported in larger European observational studies [18]. We also recognize that some of these improvements may be partly attributable to regression to the mean or the Hawthorne effect due to closer monitoring during study participation.

Beyond lipid and inflammatory control, we also found improvements in blood pressure and body weight. Mean systolic/diastolic pressures decreased from 136.9/86.3 to 126.8/79.2 mmHg, and BMI declined from 27.6 ± 3.8 to 26.3 ± 3.2 kg/m². These results suggest wider metabolic benefits of anti-TNF therapy, consistent with earlier reports of cardiometabolic improvements in patients with AS [19].

A particularly important finding was the regression of CIMT, a recognized surrogate for subclinical atherosclerosis. Right CIMT decreased from 0.84 ± 0.11 to 0.74 ± 0.09 mm and left CIMT from 0.86 ± 0.10 to 0.75 ± 0.08 mm (*P* < 0.001). While these reductions were statistically significant, their clinical meaning must be interpreted with caution. An absolute reduction of ~0.1 mm may represent improved vascular health, but does not directly confirm a reduction in clinical CV events. Long-term event-driven studies are required to determine whether such surrogate changes translate into reduced morbidity or mortality. Comparable CIMT regression has been observed in prior anti-TNF studies and attributed to improved endothelial function [20].

Multivariate regression highlighted the influence of modifiable lifestyle factors such as smoking, obesity, and physical inactivity on CV risk trajectories during therapy, consistent with previous findings [21]. These observations reinforce that pharmacologic benefits are optimized when combined with lifestyle modifications.

It is also important to acknowledge potential CV risks associated with TNF inhibitors. Although they appear to improve general CV risk profiles, evidence indicates an association with increased risk of heart failure, particularly in patients with preexisting New York Heart Association (NYHA) class III/IV disease. Emerging reports highlight that TNF inhibitors may contribute to heart failure even in younger individuals with rheumatologic conditions undergoing long-term therapy. This safety concern should inform treatment selection and monitoring [22,23].

Updated strengths and limitations

This research provides a thorough evaluation of inflammatory, metabolic, and vascular parameters in a reasonably large cohort (*n* = 246) of patients with AS over two years, offering important insights into the CV effects of anti-TNF therapy. The longitudinal design enabled assessment of both clinical and subclinical risk indicators over time. By enrolling consecutive eligible patients, employing standardized measurement protocols, and ensuring blinded CIMT assessment, the study enhances reproducibility and strengthens internal validity.

The study also has several limitations. The absence of a non-anti-TNF control group precludes direct comparison, and the observational design limits causal inference. Lifestyle factors such as diet and physical activity were self-reported, introducing possible recall bias. Dietary habits were assessed only at baseline, so their persistence or change over time could not be evaluated. Furthermore, longer-term CV outcomes (e.g., myocardial infarction or stroke) were not recorded, and follow-up was limited to one year. The single-center, convenience sampling approach may also introduce selection bias, although this was minimized by enrolling all consecutive eligible patients with uniform inclusion criteria. Additionally, by including only patients with at least three months of anti-TNF therapy, there is potential selection bias as early dropouts were excluded. Missing CIMT data in approximately 20% of patients could have introduced bias if excluded patients differed systematically from those assessed. Finally, concurrent non-biologic therapies were not systematically analyzed and may have influenced some outcomes.

Taken together, these measures mitigate some of the inherent constraints of an observational study and enhance the reliability of our findings. Future multicenter, controlled trials with longer follow-up, systematic evaluation of concomitant therapies, and inclusion of hard CV endpoints will be essential to validate these results and establish the long-term CV safety and benefits of anti-TNF therapy in AS.

## Conclusions

In patients with AS, anti-TNF therapy was associated with significant improvements in systemic inflammation and favorable changes in CV risk markers, including lipid profile, blood pressure, BMI, and CIMT. These findings underscore the potential of TNF inhibitors not only in controlling disease activity but also in mitigating CV risk, which is a major comorbidity in this population. The observed regression of subclinical atherosclerosis further supports the CV benefits of sustained inflammation control. Moreover, the influence of modifiable factors such as smoking, physical inactivity, and dietary patterns highlights the importance of integrating lifestyle interventions alongside pharmacologic therapy in order to maximize CV protection.

Future research should build on these findings through randomized controlled designs, extended follow-up, and the inclusion of hard CV outcomes such as myocardial infarction, stroke, and heart failure. Additional parameters, including endothelial function markers, advanced vascular imaging, and systematic evaluation of concomitant therapies, could provide deeper mechanistic insights. Such studies will be crucial to confirm the durability and clinical significance of the CV benefits observed and guide more comprehensive management strategies for patients with AS.
